# Mr. William John Nixon

**Published:** 1892-09-17

**Authors:** 


### HOSPITAL WORTHIES.

### MR. WILLIAM JOHN NIXON.

On Wednesday (7th inst.), at a special court of the governors of the London Hospital, the resignation of Mr. W. J. Nixon, the house governor, was received and accepted with profound regret.

For forty-six years Mr. Nixon has faithfully served the governors of the London Hospital. He was appointed secretary on July 1st, 1846, and on March 19th, 1866, house governor as well. He held both offices until June 22nd, 1875, when he relinquished the duties of secretary. For upwards of twenty-six years Mr. Nixon has had entire control of the London Hospital and of all the resident officers and servants except the chaplain and secretary. He has constantly resided at the hospital, and has had authority to suspend any officer or servant appointed by the house committee, while his powers were so great as to enable him to summon a special meeting to take into consideration the conduct of any officer appointed by a general court, at his discretion. These large powers have been exercised wisely, discreetly, and to the satisfaction not only of the committee, but of the officers and of all connected with the London Hospital. Such a record as this calls for more than a passing notice, and should win for Mr. Nixon not only the sympathy but the grateful recognition of every hospital worker throughout the world. Our earliest recollections are associated with Mr. Nixon, who brought the London Hospital into a state of efficiency far in advance of other English institutions before Miss Nightingale was heard of. We mention this fact as being the simplest method of making our readers realise the length of Mr. Nixon's services, and their importance, not only to the London Hospital, but to all the hospitals of the country.

It is not a little remarkable that under Mr. Nixon's administration, and in consequence of his ability, the London Hospital is one of the very few hospitals the administration of whose affairs are entirely vested in laymen—no member of the honorary medical staff being eligible to serve or ever having served upon any of the managing committees of this institution. Further than this, Mr. Nixon, a layman, in his capacity as house governor, has had authority, should he think the action of any medical officers, honorary or resident, calculated to be detrimental to the best interests of his institution, to suspend such officer, and of his own initiative to summon a special meeting to sit in judgment on such officer The fact that such a unique system has been in working all hese years, under Mr. Nixon, without raising a storm of opposition, or even leading to occasional disagreement, proves him to be an administrator of the first order.

The quarterly report presented at the meeting on the 7th inst. stated that the question of Mr. Nixon's resignation having arisen, a sub-committee consisting of Messrs. Ind, Hale, Carr Gomm, J. H. Buxton, and Sir Edmund Hay Currie, were appointed on July 19th to go into the whole question.

At a meeting of the sub-committee held on July 26th the following letter was received from Mr. Nixon:—

"July 21, 1892.

"To the Chairman and Members of the House "Committee.

"Gentlemen,—Influenced by consideration of my prolonged illness, and not unmindful of the age at which I have arrived, I deem it right to tender my resignation of the office of house governor.

"You will readily believe that it is not without feelings of very sincere regret, that I have come to this determination, involving as it does the severance of my connection with the London Hospital, an institution the promotion of whose interests I have endeavoured to make the main object of my life for the last forty-six years.

"Gratefully acknowledging all the kindness I have experienced at the hands of past and present members of the House Committee,

"I am, gentlemen, yours very faithfully,

"W. J. Nixon."

The following report was drawn up by the Committee:—

"That Mr. Nixon having notified to the Sub-Committee that he has tendered his resignation of the post of House Governor, your Sub-Committee feels that the reasons which have impelled him to take this step—his recent illness and his age—are such that the Committee of Governors should be recommended to accept the same.

"The Sub-Committee recommends that, as a recognition of Mr. Nixon's long and valuable services to the hospital as Secretary from July, 1846, as Secretary and House Governor from March, 1866, as House Governor from June, 1875, he should receive his full salary of £887 10s. to the end of the present year, and from that date should be paid a retiring pension of £750 a-year.

"Your Sub-Committee recommend that in future the offices of the House-Governor and Secretary shall be combined; that the officer holding this shall be styled the House-Governor, and that under him shall be the department heretofore under the control of the House-Governor and of the Secretary, and also the management of the hospital estate.

"Your Sub-Committee recommends that Mr. Roberts, the present Secretary, be recommended to the Court of Governors for election to this new office of House Governor, at a salary of £700 a year and an unfurnished house, with rates and taxes.

"It is further recommended that steps be forthwith taken to secure the services of an efficient first clerk in the Secretarial department at a salary of £250 a-year, and that Mr. Boswell continue to perform his present duties as the head of the Steward's department.

"The report of the Sub-Committee was unanimously adopted, and it was determined to recommend to the next quarterly Court of Governors, to be made special for the purpose, that Mr. Nixon's resignation be accepted.

"It was unanimously agreed that it be moved on behalf of the Committee that the Court of Governors pass a vote of thanks to Mr. Nixon in recognition of his long and valuable services to the hospital."

The Chairman in moving the adoption of the report, said that Mr. Nixon for some time past had been suffering from ill-health, and the Committee had been compelled with much regret to accept it. He had been a most loyal, most faithful, and most honourable servant of the London Hospital all these years, and they all knew that he had promoted the interests of the London Hospitals to the entire forgetfulness of his own interests.

Mr. Yatman seconded.

Sir Edmund Hay Currie said that since 1857 he had been actively connected with Mr. Nixon in his work at the London Hospital, and in all his experience he did not think he had ever come across a man who did his work so well as Mr. Nixon. He was a gentleman in every way, courteous to all, and his name was a household word in the East of London.

If to do a great work for a whole lifetime, and to do it so well that competent critics are prepared to affirm that such work could by no possibility have been done better, constitutes a claim to greatness, then Mr. Nixon may properly be called a great man. He has served two generatione with unsurpassed capacity and fidelity, and earned the grateful respect of all who know him.

